# Spatially Encoded Polaritonic Ultra‐Strong Coupling in Gradient Metasurfaces with Epsilon‐Near‐Zero Modes

**DOI:** 10.1002/adma.202510402

**Published:** 2025-09-12

**Authors:** Enrico Baù, Andreas Aigner, Jonas Biechteler, Connor Heimig, Thomas Weber, Thorsten Gölz, Stefan A. Maier, Andreas Tittl

**Affiliations:** ^1^ Chair in Hybrid Nanosystems Nano‐Institute Munich Department of Physics LMU Munich Königinstraße 10 80539 Munich Germany; ^2^ School of Physics and Astronomy Monash University Clayton Victoria 3800 Australia; ^3^ Department of Physics Imperial College London London SW7 2AZ UK

**Keywords:** bound‐states‐in‐the‐continuum, epsilon‐near‐zero, light–matter interaction, polaritonics, ultra‐strong coupling

## Abstract

A platform is introduced to achieve ultra‐strong coupling (USC) between light and matter using widely available materials. USC is a light–matter interaction regime characterized by coupling strengths exceeding 10% of the ground state energy. It gives rise to novel physical phenomena, such as efficient single‐photon coupling and quantum gates, with applications in quantum sensing, nonlinear optics, and low‐threshold lasing. Although early demonstrations in plasmonic systems have been realized, achieving USC in dielectric platforms, which offer lower losses and high Q‐factors, remains challenging due to typically low mode overlap between the photonic field and the material resonance. Here, dielectric dual gradient metasurfaces supporting quasi‐bound‐states‐in‐the‐continuum are leveraged to spatially encode both the spectral and coupling parameter space and demonstrate USC to an epsilon‐near‐zero (ENZ) mode in an ultra‐thin SiO_2_ layer. The strong out‐of‐plane electric fields in tapered bar structure overlap exceptionally well with those of the ENZ mode, resulting in a normalized coupling strength of η = 0.10 and a mode splitting equivalent to 20% of the ENZ mode energy; a four‐to‐five‐fold increase compared to previous approaches. The strong field confinement of the approach opens new possibilities for compact and scalable polaritonic devices, such as tunable frequency converters and low‐energy optical modulators.

## Graphical abstract

Dielectric dual gradient metasurfaces supporting quasi‐bound‐states‐in‐the‐continuum are leveraged to spatially encode both the spectral and coupling parameter space and demonstrate ultra‐strong coupling to an epsilon‐near‐zero mode in an ultra‐thin SiO_2_ layer.The platform achieves exceptional mode overlap, reaching a normalized coupling strength of η = 0.10 and a mode splitting equivalent to 20% of the epsilon‐near‐zero mode energy.

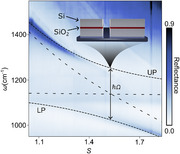

## 1 Introduction

When the interaction strength between light and matter surpasses a critical threshold, polaritons emerge as quasiparticles that combine the properties of photons with those of material excitations such as excitons or phonons.^[^ ^1^, ^2^ ^]^ These hybrid states exhibit unique characteristics, including modified dispersion relations,^[^ ^3^, ^4^ ^]^ strong optical nonlinearities,^[^ ^5^, ^6^ ^]^ and quantum coherence effects.^[^ ^7^ ^]^ First realized in microcavities,^[^ ^8^ ^]^ strong coupling (SC) has recently been observed in various nanophotonic systems,^[^ ^9^, ^10^, ^11^ ^]^ where high field confinement and small mode volumes were utilized to achieve significant coupling strengths. All‐dielectric metasurfaces provide a well‐suited platform for enhanced light‐matter interaction due to their extraordinary spectral and quality factor (Q‐ factor) tunability,^[^ ^12^, ^13^ ^]^ making them highly attractive for various applications, such as nonlinear optics,^[^ ^14^ ^]^ biosensing,^[^ ^15^ ^]^ and colorimetry.^[^ ^16^ ^]^ In particular, the high Q‐factors of dielectric metasurfaces supporting symmetry‐protected quasi‐bound‐states‐in‐the‐continuum (qBICs)^[^ ^17^, ^18^, ^19^ ^]^ allow clear observation of upper and lower polariton branches demonstrated in recent studies on SC between qBICs and plasmons,^[^ ^20^ ^]^ excitons,^[^ ^21^, ^22^, ^23^ ^]^ or molecular vibrations.^[^ ^24^, ^25^ ^]^

However, reaching higher coupling strengths would allow a wider spectral range for polaritonic effects, an increased density of photonic states, and potentially stronger nonlinear effects.^[^ ^26^ ^]^ In particular, the system can reach the ultra‐strong coupling (USC) regime when the normalized coupling strength $\eta =\frac{g}{\omega }$ reaches 0.1, where *ω* denotes the ground state frequency.^[^ ^27^ ^]^ Note that the definition of USC does not consider decay rates, but is rather characterized by the coupling strength *g*.^[^ ^28^ ^]^ While coupling in the USC regime has been successfully demonstrated in optomechanics,^[^ ^29^, ^30^, ^31^ ^]^ photochemistry,^[^ ^32^ ^]^ and 2D materials,^[^ ^33^ ^]^ achieving it in nanophotonic systems is challenging, with initial realizations exploiting plasmonic nanogaps^[^ ^34^, ^35^ ^]^ and microcavities with phononic materials.^[^ ^36^, ^37^ ^]^

In dielectric metasurfaces, including those typically used for qBICs, η has so far remained too low, around five times below the critical threshold for achieving USC,^[^ ^38^ ^]^ which can be attributed to poor mode overlap between the photonic mode and the resonant material. Unlike plasmonic systems, where strong surface fields drive efficient coupling, the fields in dielectric metasurfaces are typically more delocalized.^[^ ^12^ ^]^ Using the resonant material as a capping layer, as realized for exciton coupling with 2D materials,^[^ ^39^ ^]^ or using epsilon‐near‐zero (ENZ) materials as a substrate layer,^[^ ^40^ ^]^ has so far been insufficient to reach USC. This issue of low mode overlap is especially apparent in ENZ‐based approaches: While ENZ modes are typically confined to thin layers with characteristic out‐of‐plane field components,^[^ ^41^ ^]^ the field lines of most photonic modes are predominantly in‐plane.^[^ ^38^ ^]^ As a result, these in‐plane fields do not couple to the ENZ mode. Instead, they often couple with other intrinsic loss channels, such as transverse optical (TO) phonons, further reducing the performance of the coupled system.

Here, we present a dual‐gradient metasurface platform^[^ ^42^ ^]^ that achieves USC in the mid‐IR by coupling a dielectric qBIC with an ENZ mode. Positioning the highly subwavelength SiO_2_ layer that supports the ENZ mode at the center of tapered bar resonators enables efficient coupling to electric field vortices of the qBIC. These out‐of‐plane electric fields lead to a high mode overlap and a splitting of 28.4 meV for a 114 nm thick SiO_2_ layer, corresponding to ≈20% of the ground state frequency and a normalized coupling strength of η = 0.10; a four‐to‐five‐fold increase compared to previous approaches with similar ENZ layer thicknesses.^[^ ^38^ ^]^ The dual‐gradient metasurface design allows continuous spatial encoding of both the spectral position and the Q‐factor. We experimentally achieve mapping of the anticrossing behavior across 420 individual spectra. By leveraging the continuously tuning of the qBIC resonance from 1400 to 950 cm^−1^, our approach significantly surpasses previous coupling studies in spectral resolution, as earlier works relied on only a limited number of discrete metasurfaces to probe the anticrossing region.^[^ ^22^, ^40^, ^43^ ^]^ Furthermore, simulations show that our structure already achieves strong coupling with SiO_2_ films as thin as 5 nm, corresponding to ≈ λ_*ENZ*_/1700.

Additionally, varying the position of the SiO_2_ layer within the resonator allows to selectively control the coupling strengths of the qBIC mode to either the ENZ and TO phonon modes. With the ENZ layer placed at the center of the tapered bars, the qBIC couples exclusively to it, suppressing interaction with the spectrally close TO phonon. This selectivity contrasts with most existing systems, where the photonic mode couples to both the ENZ mode and the TO phonon,^[^ ^34^ ^]^ thereby dampening potential hybrid states in between. To our knowledge, such a level of control has not been demonstrated in any other phononic system, making our tapered bar gradient particularly well‐suited for studying polaritons in highly anisotropic media, such as layered 2D materials with interlayer excitons or phonons in thin films.

## 2 Results

**Figure 1:**
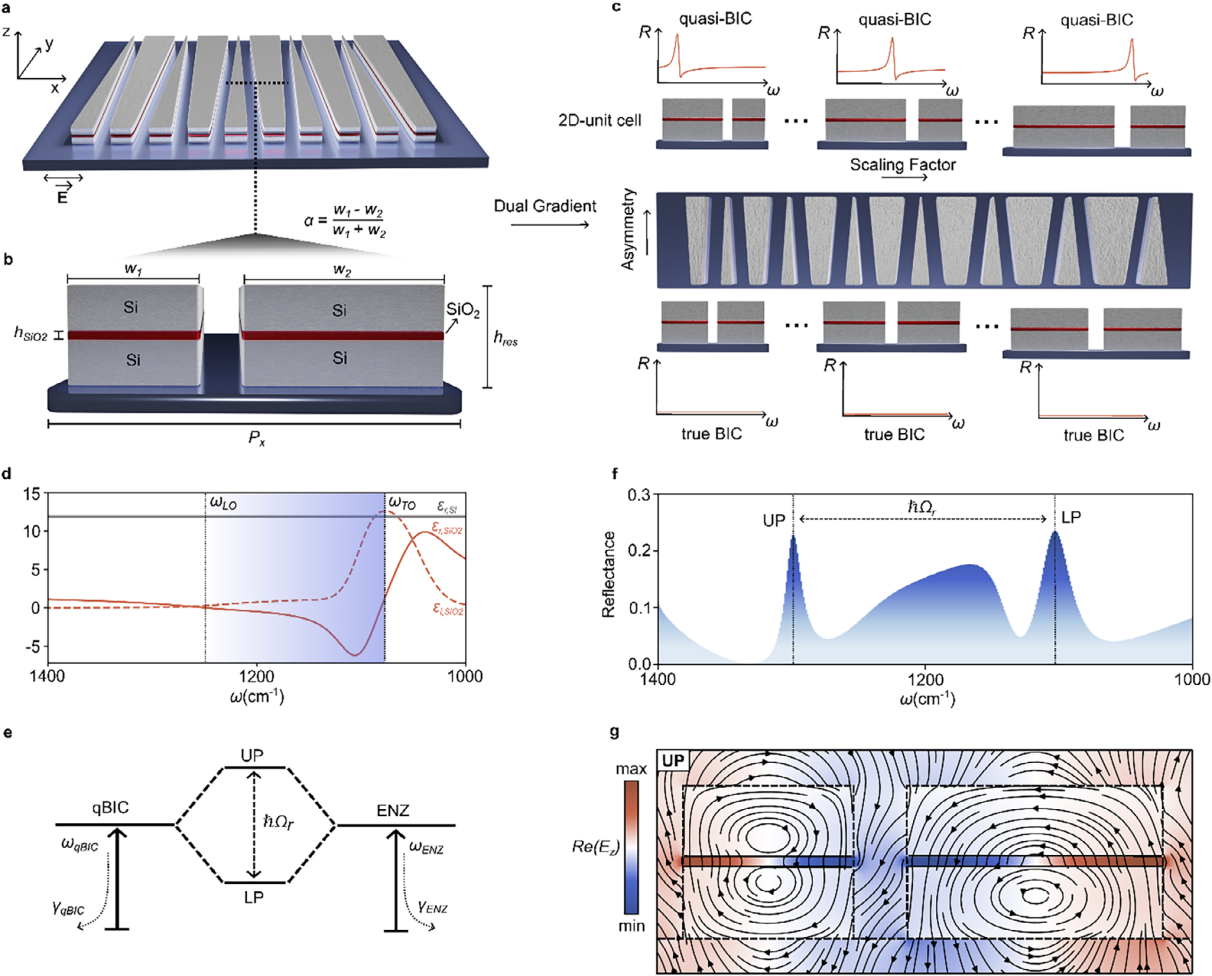
Tapered bar dual gradient for enhanced ultrastrong coupling. a) Illustration of a tapered bar metasurface gradient supporting symmetry‐protected qBIC resonances. b) Schematic of the 2D unit cell composed of Si resonators on a CaF_2_ substrate, with a thin SiO_2_ film (thickness *h*_SiO2_) sandwiched between Si layers. The pitch is given by *p*_x_, the height by *h*_res_, and the combined widths (*w*_1_ + *w*_2_) occupy 80% of *p*_x_ (80% filling factor). Their relative offset defines the asymmetry parameter α. c) Illustration of a dual‐gradient design with smoothly varying scaling and asymmetry parameters along both in‐plane axes, providing full control over the spectral position and resonance linewidth within a single metasurface. d) Real (ε_r,SiO2_, orange solid curve) and imaginary (ε_i,SiO2_, orange dashed curve) parts of the adopted SiO_2_ permittivity.^[^ ^46^ ^]^ The blue‐shaded region denotes the SiO_2_ reststrahlen band (ε_i_ < 0). The solid gray curve shows the permittivity of Si, and the TO and LO phonon frequencies are indicated at ω_TO_ and ω_LO_, respectively. e) Schematic illustrating the strong coupling between a qBIC and an ENZ mode, resulting in a Rabi splitting ℏΩ_r_. f) Simulated spectrum showing the splitting of the qBIC mode into UP and LP for *h*_SiO2_  = 80 nm. g) Simulated out‐of‐plane electric field *E*_z_ and field lines (black arrows) within a single unit cell at the UP frequency, revealing strong field confinement in the resonant SiO_2_ layer.

The proposed dual‐gradient metasurface consists of continuously tapered Si bars on a CaF_2_ substrate (**Figure** **1**a). As illustrated in Figure 1b, the unit cell has a pitch *p*_x_ along the *x*‐axis and contains two bars of height *h*_res_ aligned along the *y*‐axis with widths *w*_1_ and *w*_2_ with *w*_1_ + *w*_2_ = 0.8 *p*_x_. Because the bars continuously span along the whole *y*‐axis of the gradient, the *y*‐pitch *p*_y_ can be considered infinitesimal. A thin SiO_2_ layer of thickness *h*_SiO2_ serves as the resonant material and is placed at the center of the tapered bars to maximize coupling strength. To break the symmetry and convert the true BIC into a measurable qBIC with a finite Q‐factor, an offset between *w*_1_ and *w*_2_ is introduced. Increasing this offset decreases the radiative Q‐factor *Q*_rad_, following the characteristic relation for symmetry‐protected qBICs, *Q*_rad_∝1/α^2^, where the asymmetry parameter α is defined as the relative width offset

(1) $$\alpha =\frac{w_{1}-w_{2}}{w_{1}+w_{2}}$$

We select x‐polarized light normal to the tapered bars due to the advantageous mode profile of this qBIC. To map both the spectral and coupling space around the ENZ mode, we design dual‐gradient metasurfaces (Figure 1c), where the unit cell size continuously increases along the *x*‐axis with a scaling factor *S* to achieve broad spectral coverage. Additionally, by increasing the asymmetry along the *y*‐axis, the radiative Q‐factor can be arbitrarily tuned, limited only by intrinsic losses and fabrication imperfections.

Compared to previous dual gradient or other high Q‐factor gradient designs,^[^ ^42^, ^44^, ^45^ ^]^ our unit cell geometry overcomes a key limitation: the misalignment of neighboring unit cells along the scaling direction. In conventional designs, the scaling creates a size mismatch between adjacent unit cells, leading to misalignment between the unit cells, which has been shown to degrade the gradient's performance.^[^ ^42^ ^]^ Our tapered bar geometry prevents this effect due to the infinitesimal *p*_y_, ensuring perfect alignment for neighboring unit cells (see Figure S1, Supporting Information).

The target wavelength range is set by the dielectric function of SiO_2_ (Figure 1d), which features a TO phonon and a longitudinal optical (LO) phonon in close proximity to the ENZ region.^[^ ^46^ ^]^ When the gradient is properly scaled, the qBIC and the ENZ mode couple to form an upper polariton (UP) and a lower polariton (LP), separated by the Rabi splitting ℏΩ_r_, as depicted in the energy level diagram in Figure 1e. An exemplary numerical spectrum for a SiO_2_ layer with *h*_*SiO*2_ = 80 nm is shown in Figure 1f and reveals three distinct modes: the UP on the left, the LP on the right, and a broad ENZ mode in between. The asymmetric profile of the ENZ mode can be attributed to the dielectric function of SiO_2_, which has a small feature ≈ 1200 cm^−1^ due to an asymmetric tension of the out‐of‐phase Si–O–Si mode.^[^ ^47^ ^]^ Figure 1g shows the electric field distribution at the UP branch of Figure 1f. The field lines form two vortices of opposite handedness within the tapered bars, revealing the two antiparallel magnetic dipoles characteristic of the qBIC. We verify that the magnetic dipole is the main contributor to the qBIC mode through multipole decomposition (Figure S2, and Note S2, Supporting Information). The color‐coded *E*_z_ component highlights strong out‐of‐plane fields within the SiO_2_ layer, characteristic of ENZ modes. Hence, the observed state clearly combines the features of both the qBIC and ENZ modes, signifying polaritonic behavior.

**Figure 2:**
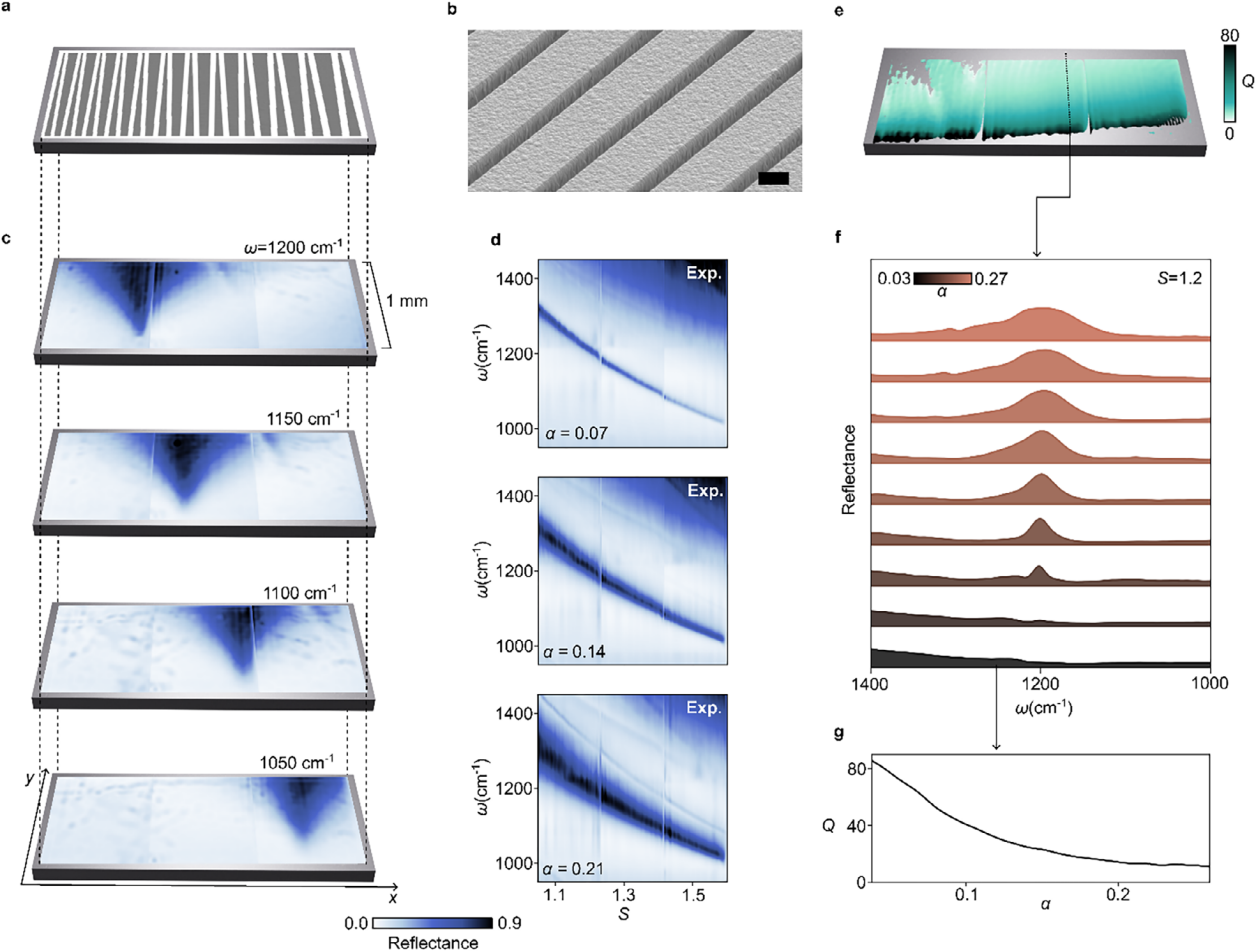
Optical characterization of the tapered bar dual gradient. a). Schematic of the dual‐gradient design. b). SEM image of the fabricated metasurface in tilted view. Scale bar: 1μm. c). Reflectance snapshots of the gradient at four selected wavelengths, where high‐reflectance regions indicate a resonance. d). Reflectance spectra vs scaling factor S, extracted along the *x*‐axis of the dual gradient for different asymmetry parameters. For comparison, simulations are shown in Figure S6 and S7 (Supporting Information). e). Q‐factor map, with each pixel individually fitted. f). Reflectance spectra extracted along the y‐cut shown in (f). g). Corresponding Q‐factors.

Before introducing resonant SiO_2_ into the gradient, we experimentally analyze pure Si gradient metasurfaces. A Si layer with a height of 1400 nm was patterned using electron beam lithography to create a 1  ×  3 mm^2^ gradient, as depicted in **Figure** **2**a. The scaling factor *S*, and thus the spectral encoding, varies along the *x*‐axis, while the *y*‐axis encodes asymmetries ranging from symmetric at the bottom to highly asymmetric at the top. Figure 2b shows a tilted view scanning electron microscope image for the symmetric and asymmetric cases, respectively.

All measurements were performed using a spectral imaging setup with quantum cascade laser sources (see Methods and Figure S3, Supporting Information). Snapshots of the gradient at four distinct wavelengths, captured in reflection mode, are presented in Figure 2c. The resonant areas of the dual gradient (visible as regions of high reflection) show a clear shift toward higher scaling factors along the *x*‐axis as the wavenumber decreases. Additionally, along the *y*‐axis, the high‐reflectance region broadens, reflecting the decreasing Q‐factor of the qBIC mode with increasing asymmetry factor α. The spectra for all *S* values, measured at three distinct α, are shown in Figure 2d. The mode broadens as α increases from 0.07 to 0.21, while the gradient continuously shifts the resonance frequency. Here, *S* = 1 corresponds to a pitch of *p*_x_ = 4000 nm. We note that the wavelength is not shifting perfectly linear with *S*, because the resonator height is constant across the entire gradient and thus the mode volume does not scale linearly with *S* (Figure S4, Supporting Information). Mapping the Q‐factor for each individual pixel reveals the Q‐factor map in Figure 2e, with spectra taken from the *y*‐axis cut shown in Figure 2f. Their corresponding Q‐factors are shown in Figure 2g smoothly decreases with α from ≈ 80 to less than 20. The corresponding characteristic antiparallel mode profile is shown in Figure S5 (Supporting Information).

**Figure 3:**
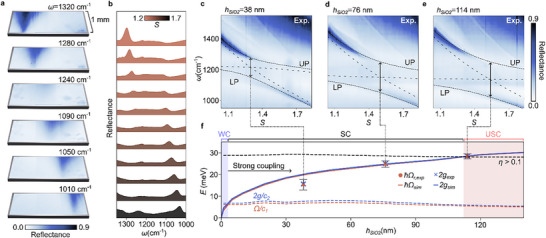
Experimental ultrastrong coupling between qBIC and ENZ mode. a). Reflectance images of the metasurface at six different wavelengths, illustrating the strong coupling behavior. b). Extracted reflectance spectra for varying scaling factors and an asymmetry parameter of α = 0.15, showing splitting of the qBIC mode around the ENZ mode. c–e). Reflectance spectra for SiO_2_ film thicknesses of *h*_*SiO*2_ = 38, 76, and 114 nm, respectively, showing a clear splitting of the qBIC mode into the upper (UP) and lower (LP) polaritons around the ENZ mode, which increases with film thickness. f). Simulated ℏΩ_r,sim_ (solid orange curve) and measured ℏΩ_r,exp_ (orange dots) for the Rabi splitting, as well as the coupling strengths *g*_sim_ (solid blue curve) and *g*_exp_ (blue crosses), plotted vs film thickness to highlight enhanced coupling for thicker films. The dashed orange and blue curves represent the strong coupling conditions *c*_1_ and *c*_2_, while the dashed black line marks the USC regime where η > 0.1. The blue and red regions delineate the WC and USC regimes, respectively.

Next, we insert the SiO_2_ layer into our design at the center of the Si tapered bars. To probe the coupling between the qBIC and the ENZ mode, we conduct reflectance measurements. The resulting single‐wavelength images (**Figure** **3**a) for *h*_*SiO*2_ = 38 nm, well below the λ/50 limit in which ENZ modes are excited,^[^ ^41^ ^]^ reveals the coupling when compared sequentially: When approaching the ENZ region, the qBIC disappears due to the energy gap between both polaritonic branches. The qBIC resonance then reappears ≈ 1100 cm^−1^. This splitting is also evident in the individual spectra plotted in Figur 3b. To determine the coupling parameters of this anticrossing behavior, we perform measurements of three dual gradients with different SiO_2_ thicknesses. The spectrally resolved scaling sweeps for *h*_*SiO*2_ = 38 , 76 , and 114 nm are shown in Figure 3c–e, respectively. The resulting hybrid modes are fitted (dashed lines) using the well‐known coupled‐oscillator model, for which the Hamiltonian *H*_2_ is given by

(2) $$H_{2}=\hbar \left(\begin{array}{cc} \omega_{\mathrm{qBIC}}-i\gamma_{\mathrm{qBIC}} & g\\ g & \omega_{\mathrm{ENZ}}-i\gamma_{\mathrm{ENZ}} \end{array}\right)$$

where ω_qBIC_ and ω_ENZ_ are the spectral positions and γ_qBIC_ and γ_ENZ_ are the loss rates of the two resonances, respectively. The loss rates used for the following calculations were fitted from the experimental data with a Fano lineshape fit (see Figure S8, Supporting Information).

By fitting the eigenvalues of *H*_2_ to the UP and LP branches (see Note S1, Supporting Information), we extract the coupling strengths *g* and the Rabi splitting ℏΩ_r_. Note that while for most SC processes, the spectral position of the polaritonic resonance remains fixed, here, the ENZ mode may shift when *S* increases because of geometrical alterations, such as changes in resonator width. To account for the non‐linear scaling of the qBIC frequency with *S*, we fit both the spectral positions and the linewidths from simulations using the respective film thicknesses, and then incorporate these results into our model. This approach yields more accurate fits by including the complex dielectric function of SiO_2_. The experimental results for all three SiO_2_ layer thicknesses are shown as points (Table S1, Supporting Information) in Figure 3f, alongside the simulated results (solid line, Table S2, Supporting Information), which are shown in Figure S9 (Supporting Information). Both sets agree well and show an increasing coupling with SiO_2_ thickness. To verify whether the measured gradients exhibit SC, we evaluate two standard criteria, *c*_1_ and *c*_2_, where *c*_1_ = Ω_r_/(γ_qBIC_ + γ_ENZ_) > 1 and $c_{2}=g/\sqrt{(\gamma_{qBIC}^{2}+\gamma_{ENZ}^{2})/2}/>1$.

Both criteria, plotted as dashed lines in Figure 3f, are satisfied by all measurements. Notably, for the thickest measured film (*t* = 114 nm), *c*_1_ = 5.4 and *c*_2_ =  5.0 correspond to a remarkably large Rabi splitting of ℏΩ_r_ = 28.4 meV. This is equivalent to ≈20% of the ENZ mode energy, yielding η = 0.10, which lies within the USC regime. In simulations, the USC regime is clearly reached for film thicknesses of *h*_*SiO*2_ = 120 nm and *h*_*SiO*2_ = 140 nm. In principle, the coupling strengths achievable in our platform could be further enhanced by increasing the SiO_2_ layer thickness. However, we note that both polariton branches experience stronger absorption for larger thicknesses of SiO_2,_ and the condition of *h*_*SiO*2_ <  λ_*ENZ*_/50 should always be fulfilled for the excitation of ENZ modes. This would limit the maximum layer thickness to ≈ 170 nm for our system. Remarkably, in simulations, the transition from weak to strong coupling already occurs for *h*_*SiO*2_ = 5 nm, corresponding to ≈λ_*ENZ*_/1700.

**Figure 4:**
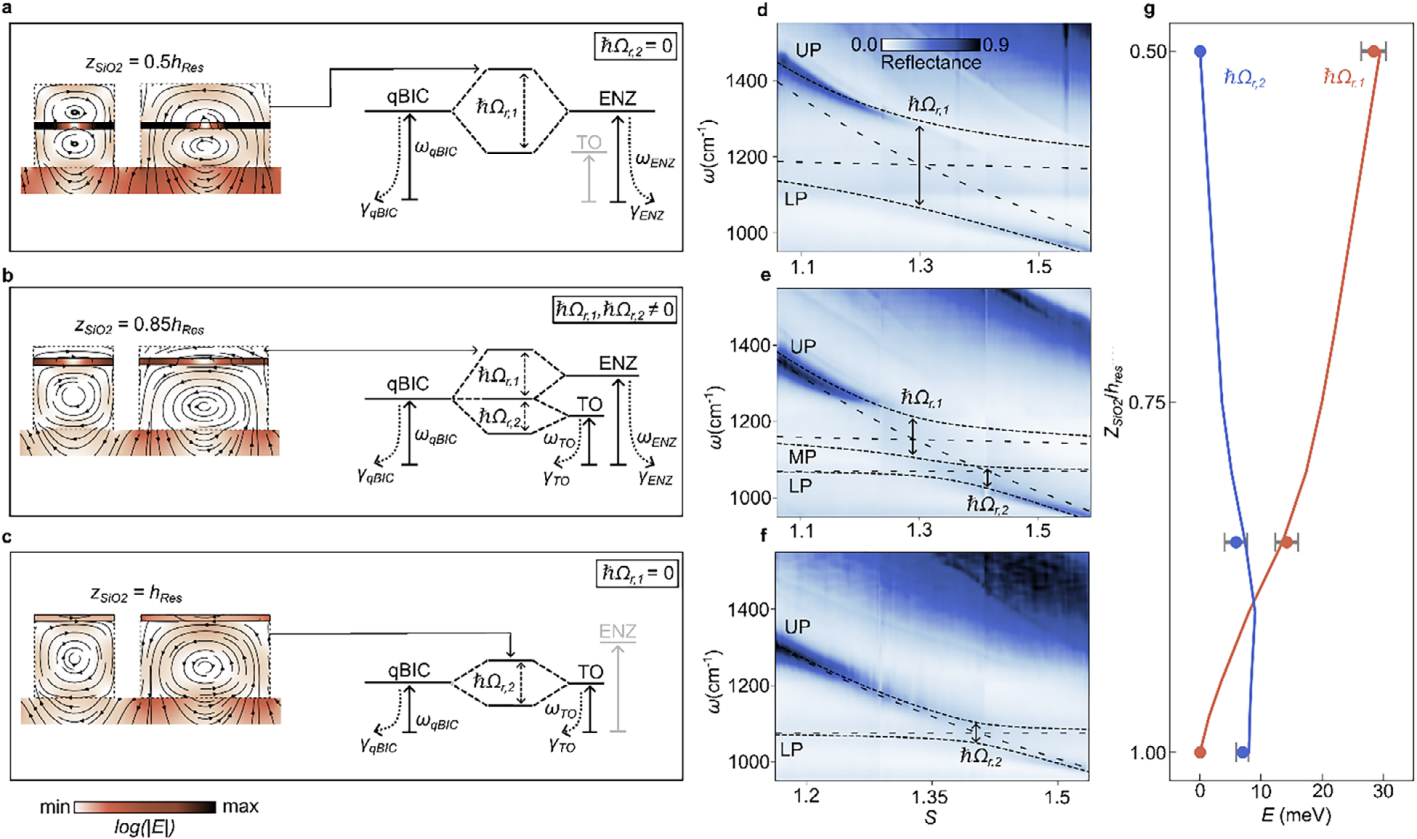
Experimental realization of selective strong coupling to the TO phonon and ENZ mode. a–c). Simulated electric field distributions for different SiO_2_ film positions and corresponding strong coupling diagrams. a). When the film is centered, ℏΩ_r,2_ = 0, indicating no qBIC–TO phonon coupling. b). At intermediate positions (*z*_SiO2_/*h*_res_ = 0.85), both the ENZ mode and TO phonon couple to the qBIC and $\hbar \Omega_{r,2}\neq 0$. c). Moving the film to the top (*z*_SiO2_/*h*_res_ = 1) negates the ENZ mode influence and ℏΩ_r_ = 0, allowing pure qBIC–TO phonon coupling. d–f). Experimental demonstration of strong coupling for (d) qBIC–ENZ, (e) qBIC–ENZ‐TO and (f) qBIC–TO. Blow‐up spectra extracted from (e) are shown in Figure S11 (Supporting Information). g). Simulated (solid curves) and measured (dots) Rabi splittings for qBIC–ENZ (orange) and qBIC–TO (blue), plotted as a function of the SiO_2_ film position.

In these measurements, no energy splitting or absorption was observed at the spectral position of the TO phonon (located ≈ 1080 cm^−1^)^[^ ^46^ ^]^ clearly indicating that the TO phonon does not interact with the qBIC mode when the SiO_2_ layer is at the center of the tapered bar. This selective coupling is a result of the qBIC mode profile, first discussed in Figure 1g. There, the two antiparallel magnetic dipoles form an electric field vortex within each tapered bar. Crucially, at the resonator center, in‐plane electric fields are nearly absent and the out‐of‐plane components align perfectly with the confinement‐axis of the thin SiO_2_ layer. Since the atomic displacement for TO phonons is parallel to *E*_z_, it cannot couple to the vortex, resulting in a complete suppression of qBIC–TO coupling (**Figure** **4**a). In contrast, the LO phonons can couple effectively to the qBIC because their atomic displacement is perpendicular to *E*_z_.^[^ ^41^, ^48^ ^]^ The electric field vortex within the tapered bars offers unique possibilities for controlling the coupling between the qBIC and the phonon modes. When the SiO_2_ film is positioned off‐center (Figure 4b), both in‐plane and out‐of‐plane electric fields are present, enabling coupling to both the ENZ and TO phonon modes. By following the circular field lines within the tapered bars, it becomes clear that shifting the film away from the center enhances coupling to the TO phonon while reducing interaction with the ENZ mode. When the film is positioned at the very top of the bars (Figure 4c), ENZ coupling is strongly suppressed as the electric field orientation and the confinement axis are perpendicular to each another, allowing only the TO phonon to interact with the qBIC. We can describe this situation using a three‐oscillator model with a Hamiltonian *H*_3_ of the form

(3) $$H_{3}=\hbar \left(\begin{array}{ccc} \omega_{\mathrm{qBIC}}-i\gamma_{\mathrm{qBIC}} & g_{1} & g_{2}\\ g_{1} & \omega_{\mathrm{ENZ}}-i\gamma_{\mathrm{ENZ}} & 0\\ g_{2} & 0 & \omega_{\mathrm{TO}}-i\gamma_{\mathrm{TO}} \end{array}\right)$$

where ω_qBIC_, ω_ENZ_, and ω_TO_ denote the resonant frequencies and γ_qBIC_, γ_ENZ_, and γ_TO_ are the corresponding loss rates. Here, *g*_1_ and *g*_2_ are the coupling strengths, and ℏΩ_r,1_ and ℏΩ_r,2_ are the respective Rabi splittings for the qBIC–ENZ and qBIC–TO phonon modes. The coupling strengths *g*_1_ and *g*_2_ can be calculated from the Rabi splittings and losses of each individual mode (see Note S1, Supporting Information). Interactions between the ENZ and TO phonon modes are assumed to be negligible.

Figure 4d–f present the corresponding experimental data for various SiO_2_ film positions. For *z*_SiO2_/*h*_res_ = 0.5 and *z*_SiO2_/*h*_res_ = 1, we observe a full suppression of the coupling to the TO phonon and ENZ mode, respectively. We obtain an energy splitting of ℏΩ_r,1_ = 28.4 meV (η = 0.10) for *z*_SiO2_/*h*_res_ = 0.5 (Figure 4d) and ℏΩ_r,2_ = 7.0 meV (η = 0.03) for *z*_SiO2_/*h*_res_ = 1 (Figure 4f). In the case of *z*_SiO2_/*h*_res_ = 0.85, the qBIC mode couples to both the ENZ mode and TO phonon, yielding ℏΩ_r,1_ = 14.2 meV (η = 0.06) and ℏΩ_r,2_ = 5.9 meV (η = 0.02) (Figure 4e).

Notably, while energy splittings on both modes can be observed experimentally, fully resolving all three energy levels with LP, middle polariton (MP), and UP is challenging due to the relatively high losses in SiO_2_. These losses could be mitigated by using alternative phonon‐polaritonic materials such as SiC or hBN, which exhibit lower dissipation. The simulated energy splittings (Figure 4g; Table S3, Supporting Information), together with experimental data (Table S4, Supporting Information), show that ℏΩ_r,1_ drops from 28.4meV to 0meV when the SiO_2_ position is shifted toward the top of the silicon layer. In contrast, ℏΩ_r,2_ increases, reaching a maximum splitting of 9.0meV at *z*_SiO2_/*h*_res_ = 0.9, as confirmed by numerical simulation results in Figure S10 (Supporting Information).

Finally, we can maximize the coupling between the SiO_2_ layer and the qBIC by designing a new gradient structure that efficiently interacts with both the TO phonon and the ENZ mode. This gradient is equivalent to the one shown in Figure 3 but includes an additional SiO_2_ layer at the top, as illustrated in Figure S12 (Supporting Information). The resulting coupling can be described by both the TO and ENZ modes contributing to an energy splitting of ℏΩ_r,1, 2_ = 29.6 meV (Figure S12b, Supporting Information). While the UP and LP are clearly visible, no MP appears. This is likely caused by the dominant qBIC–ENZ coupling, which hybridizes the resonance beyond the spectral position of the TO phonon. Nevertheless, the observed energy splitting remains significantly larger than the pure qBIC–ENZ mode, indicating the influence of the TO phonon. The coupling strength *g* achieved with this design exceeds 20% of the uncoupled energies of the system, pushing even further into the USC regime.

## 3 Discussion

We successfully demonstrate ultrastrong coupling between an ENZ mode and a qBIC resonance in film thicknesses below the λ/50 limit in which ENZ modes can still be excited.^[^ ^41^ ^]^ This extraordinary coupling is made possible by the strong mode overlap between the qBIC and the ENZ mode, outperforming previous designs, as compared in Figure S13 (Supporting Information). Simulations even indicate that a thickness of 5 nm is sufficient to enter the SC regime, much smaller than in previous designs and deeply subwavelength (*h*_*SiO*2_ < λ_*ENZ*_/1700). Our dual‐gradient metasurface continuously encodes the Q‐factor and spectral position, enabling SC measurements with unprecedented experimental precision. Furthermore, integrating this tapered bar design into gradients resolves misalignment issues between neighboring unit cells, which previously limited the performance of comparable gradient approaches.^[^ ^42^, ^44^, ^45^ ^]^ Moreover, these gradients are readily transferable to other spectral regions, including the near‐IR, where nonlinear effects in materials such as indium tin oxide (ITO) can be utilized, or the visible range of light, where excitons in 2D materials can be observed. In particular, the practical realization of SC between qBICs and 2D materials in thin flakes can be challenging using conventional platforms due to the limited size of exfoliated flakes for many materials. In contrast, our continuous gradient could enable the realization of SC within a single flake by covering the full coupling parameter space.

Furthermore, the combination of extremely small mode volumes and low‐loss dielectrics sets our qBIC concept apart from both conventional dielectric and plasmonic implementations of nonlinear ENZ systems: Dielectric designs often exhibit weaker field confinement, while plasmonic systems experience higher losses that diminish the fraction of energy available for nonlinear processes. Such an increase in experimentally achieved coupling strengths paves the way for further exploration of the SC regime and potentially even the deep strong coupling regime.^[^ ^49^, ^50^ ^]^ The performance of our platform could be boosted substantially by designing qBICs with higher Q‐factors, since this would lead to an increase in coupling strengths, as has been demonstrated in previous studies.^[^ ^18^, ^40^, ^48^ ^]^ This would likely require the use of materials with higher oscillator strengths and lower losses within the Reststrahlenband, such as hBN or SiC. Thus, the large splitting and small mode volume offer ideal conditions for active optical tuning of electrically gated ITO,^[^ ^51^, ^52^, ^53^ ^]^ potentially enabling broad spectral tunability and control over the SC regime. To illustrate how such a device would function, we performed simulations using a thin layer of ITO instead of SiO_2_ (Figure S14, Supporting Information) and tuned the carrier density in the ITO film. This leads to a spectral shift of the plasma frequency and thus enables an electrically tunable ENZ mode. This mode can be coupled to a qBIC resonance tailored for the near‐infrared region of light, leading to the formation of UP and LP branches. Our simulations show that a film of 5 nm is already enough to achieve significant splitting of the qBIC mode, enabling a spectrally tunable qBIC resonance that can be spectrally shifted ≈10–15%.

Lastly, we demonstrate selective coupling of qBICs to either the TO or ENZ mode, a unique feature of our design. We can experimentally suppress either the TO phonon or the ENZ contribution simply by adjusting the position of the film within the resonator. This strategy is valuable not only for phononic materials but also for transition metal dichalcogenides (TMDCs) to isolate specific excitonic resonances or interband transitions^[^ ^54^ ^]^. Crucially, the distinctive and by height‐tunable field distribution in the resonator could facilitate the generation of interlayer excitons^[^ ^55^ ^]^ in stacked TMDC metasurfaces, which require strong out‐of‐plane electric field enhancements for efficient excitation. Additionally, the controlled suppression of specific modes could enable selective probing of SC between qBICs and anisotropic materials that have overlapping Reststrahlenbands, such as YVO_4_,^[^ ^56^ ^]^ potentially unlocking the ability to control the propagation of hyperbolic polaritons via the photonic mode. Finally, the excellent mode overlap and high field confinement achieved in our platform offer a direct pathway toward the realization of tunable frequency converters and optical modulators, which may require strong nonlinear effects to function efficiently.

## 4 Experimental Section

### Numerical Methods

The simulations were conducted using CST Studio Suite (Simulia), a commercial finite element solver. The setup included adaptive mesh refinement and periodic boundary conditions in the frequency domain. SiO_2_ was modeled according to the data provided by Franta et al.,^[^ ^46^ ^]^ and the refractive indices of Si and CaF_2_ were set to 3.32 and 1.43, respectively.

### Fabrication

For the coupled qBIC‐ENZ gradients, fabrication began with the sequential deposition of amorphous silicon via plasma‐enhanced chemical vapor deposition (PECVD) using a PlasmaPro 100 system (Oxford Instruments) with a thickness‐error of ≈±5 nm. This was followed by radio frequency sputtering of SiO_2_ using an Amod PVD system (Angstrom Engineering) and a second silicon deposition step. Finally, a chromium layer was sputtered using the Amod PVD system. The films were spin‐coated with a positive electron beam resist, CSAR 62 (Allresist), and patterned via electron beam lithography using an eLINE Plus system (Raith) at 20 kV with a 30 µm aperture. The developed patterns were processed in an amyl acetate bath, followed by a MIBK:IPA (1:9 ratio) bath. Reactive ion etching (RIE) was performed using the PlasmaPro 100 system (Oxford Instruments) to selectively etch the film layers in the following order: chromium (serving as a hard mask after the resist layer was removed), silicon, SiO_2_, silicon, and finally chromium to strip the remaining hard mask. The pure silicon gradient, the gradient with SiO_2_ on top, and the version with silicon both on top and at the center were fabricated following the same procedure, with adjustments only to the deposition and etching sequence. The fabrication workflow is visualized in Figure S15 (Supporting Information).

### Optical Characterization

Optical measurements were conducted using a Spero mid‐infrared spectral imaging microscope (Daylight Solutions Inc.), as shown in Figure S3 (Supporting Information). The system included a 4× magnification objective (NA = 0.15), providing a 2 mm^2^ field of view with a resolution of 480 × 480 pixels and a pixel size of ≈4 × 4 µm^2^. It was equipped with three tunable quantum cascade lasers, covering wavelengths from 5.6 to 10.5 µm with a spectral resolution of 2 cm^−1^. The lasers emitted linearly polarized light, which was crucial for our measurements. To capture the full 1 × 3 mm^2^ gradient, three spectral images were acquired and stitched together during post‐processing.

## Conflict of Interest

The authors declare no conflict of interest.

## Author Contributions

E.B., A.A., and J.B. contributed equally to this work. E.B., A.A., and A.T. conceived the idea and planned the research. A.A., J.B., and C.H. contributed to the sample fabrication. E.B., A.A., J.B., and T.G. performed the measurements. E.B. conducted the numerical simulations. E.B., A.A., J.B., and T.W. contributed to the data processing. E.B., A.A., J.B., T.W., S.A.M., and A.T. contributed to the data analysis. S.A.M. and A.T. supervised the project. All authors contributed to the writing of the paper.

## Supporting information



Supporting Information

Supplementary Movie S1

Supplementary Movie S2

Supplementary Movie S3

## Data Availability

The data that support the findings of this study are openly available in Zenodo at https://doi.org/10.5281/zenodo.16812941, reference number 1681294.
