# Supplementary material for: Spatially Encoded Polaritonic Ultra‐Strong Coupling in Gradient Metasurfaces with Epsilon‐Near‐Zero Modes
Source: Adv Mater. 2025 Sep 12;38(1):e10402. doi: 10.1002/adma.202510402 (PMC12759211; doi:10.1002/adma.202510402)
Supplement: Supplementary file 1 — Supporting Information [file ADMA-38-e10402-s002.docx]

**Supporting Information**

**Spatially Encoded Polaritonic Ultra-Strong Coupling in Gradient Metasurfaces with Epsilon-Near-Zero-Modes**

*Enrico Baù^†^, Andreas Aigner^†^, Jonas Biechteler^†^, Connor Heimig, Thomas Weber, Thorsten Gölz, Stefan A. Maier, and Andreas Tittl**

^†^These authors contributed equally to this work

*Corresponding Author

**Supplementary Note 1: Coupled oscillator model**

To determine the coupling strengths from simulated and experimental data, a coupled oscillator model was utilized. Since the contribution of the TO phonon can be ignored when the SiO_2_ layer is sandwiched between the resonator material, the system studied in **Fig. 3** can be described by a 2x2 Hamiltonian *H_2_* (as shown in the main manuscript). By diagonalizing *H_2_*, we can obtain two distinct parabolic branches *ω_±_* which describe the spectral positions of both hybrid states, called upper polariton and lower polariton:

$$\omega_{\pm}= \frac{\omega_{qBIC}+\omega_{ENZ}}{2}+ \frac{i(\gamma_{qBIC}+ \gamma_{ENZ})}{2} \pm\sqrt{g^{2}-\frac{1}{4}(\gamma_{qBIC}-\gamma_{ENZ}+i{(\omega_{qBIC}-\omega_{ENZ}))}^{2}}$$

We fitted this expression to results and simulations shown in **Fig. 3** to retrieve the coupling strength *g.* At zero detuning ($\omega_{qBIC}=\omega_{ENZ})$_,_ the rabi splitting can be obtained by the following expression:

$$\Omega_{r}= 2\sqrt{g^{2}-\frac{1}{4}(\gamma_{qBIC}-\gamma_{ENZ})^{2}}$$

To study the coupling between TO phonon, ENZ mode and qBIC mode shown in **Fig.4**, we utilized a three harmonic oscillator model, described by a Hamiltonian *H_3_*. The procedure to determine the two coupling strengths *g_1_* between qBIC and ENZ mode and *g_2_* between qBIC and TO phonon is equivalent to the two coupled oscillator model shown above, except that three parabolic branches (UP, MP and LP) are fitted to extract all coupling strengths simultaneously.

**Supplementary Note 2: Multipole decomposition**

To gain deeper insights into the physical origin of our quasi-BICs, we perform a multipole decomposition of the current density

$$\mathbf{J}=-i\omega\epsilon_{0}\left( \epsilon_{r}-\epsilon\right)\mathbf{E}$$

inside the resonator, where $\mathbf{E}$ is the electric field, $\epsilon_{r}$ and $\epsilon$ the relative permittivity of the resonator material and the environment, respectively, $\epsilon_{0}$ is the vaccum permittivity and $\omega$ the frequency. The multipolar components are spatial integrals over the current density with definitions taken from.^[1]^

We can then write the scattered electric field as

$$\mathbf{E}_{\mathrm{scat}}\left( \mathbf{r} \right)\mathbf{=}\frac{k_{0}^{2}e^{ikr}}{4\pi\epsilon_{0}r}\left( \left[ \mathbf{n}\times\left[ \mathbf{p}\times\mathbf{n} \right] \right]\boldsymbol{+}\frac{1}{\nu}\left[ \mathbf{m}\times\mathbf{n} \right]+\frac{ik}{2}\left[ \mathbf{n}\times\left[ \mathbf{n}\times\hat{Q}_{e}\mathbf{n} \right] \right]+\frac{ik}{2\nu}\left[ \mathbf{n}\times\hat{Q}_{m}\mathbf{n} \right] \right)$$

where **p** denotes the electric dipole moment (ED), **m** the magnetic dipole (MD) and $\hat{Q}_{e}$ the electric and $\hat{Q}_{m}$ the magnetic quadrupole tensors. To study the composition of multipoles, we evaluate the contribution of the individual multipoles to the total scattered power

$$P_{\mathrm{scat}}=\frac{1}{2}\sqrt{\frac{\epsilon_{0}\epsilon}{\mu_{0}}}\int\left| \mathbf{E}_{\mathrm{scat}} \right|^{2}d\Omega$$

As the periodicity of the metasurface and the excitation conditions further limit scattering to the far field, we can reconstruct the reflectance spectrum purely from near-field analysis, where the reflection parameter for excitation in x-direction is given by^[2]^

$$r=\frac{ik}{E_{0}2S_{L}\epsilon_{0}\epsilon}\left( p_{x}-\frac{1}{v}m_{y}+\frac{ik_{d}}{6}Q_{xz}^{e}-\frac{ik_{d}}{2v}Q_{xz}^{m} \right)$$

Here, $k=k_{0}\sqrt{\epsilon}$ is the wave number and $v=1/\sqrt{\mu_{0}\epsilon_{0}\epsilon}$ is the speed of light in the surrounding medium. $E_{0}$ is the magnitude of the exciting electric field and $S_{L}$ the area of a unit cell. The reflectance is calculated via $R=\left| r \right|^{2}$.

**Supplementary Tables**

| *h_SiO2_*(nm) | *Ω_r_*(meV) | *g*(meV) | *ω_ENZ_*(cm^-1^) | *γ_ENZ_*(cm^-1^) | *η* |
| --- | --- | --- | --- | --- | --- |
| 38 | 15.2±2.5 | 7.8±1.5 | 1182 | 41 | 0.05±0.01 |
| 76 | 24.8±1.5 | 12.5±1.1 | 1152 | 39 | 0.09±0.01 |
| 114 | 28.4±1.2 | 14.2±0.8 | 1135 | 31 | 0.101+0.01 |

**Table S1: Experimental coupling strengths and energy splittings for various SiO2 layer thicknesses.**

| *h_SiO2_*(nm) | *Ω_r_*(meV) | *g*(meV) | *ω_ENZ_*(cm^-1^) | *γ_ENZ_*(cm^-1^) | *η* |
| --- | --- | --- | --- | --- | --- |
| 1 | 3.4 | 2.2 | 1164 | 36 | 0.016 |
| 3 | 5.9 | 3.3 | 1164 | 36 | 0.023 |
| 5 | 8.3 | 4.4 | 1164 | 36 | 0.030 |
| 10 | 11.1 | 5.7 | 1164 | 36 | 0.040 |
| 20 | 15.2 | 7.8 | 1168 | 42 | 0.054 |
| 30 | 18.2 | 9.3 | 1184 | 39 | 0.063 |
| 40 | 20.1 | 10.2 | 1181 | 42 | 0.070 |
| 50 | 21.7 | 11.0 | 1173 | 44 | 0.076 |
| 60 | 22.8 | 11.6 | 1164 | 43 | 0.080 |
| 80 | 25.0 | 12.6 | 1149 | 39 | 0.088 |
| 100 | 26.7 | 13.4 | 1139 | 33 | 0.095 |
| 120 | 29.1 | 14.6 | 1133 | 30 | 0.104 |
| 140 | 30.1 | 15.1 | 1129 | 28 | 0.108 |

**Table S2: Simulated coupling strengths, energy splittings and decay rates for various SiO2 layer thicknesses.**

| *z_SiO2_/h_res_* | *Ω_r,1_*(meV) | *Ω_r,2_*(meV) | *g_1_*(meV) | *g_2_*(meV) |
| --- | --- | --- | --- | --- |
| 0.5 | 29.5 | 0 | 14.8 | 0 |
| 0.7 | 22.1 | 2.9 | 11.4 | 2.0 |
| 0.75 | 20.0 | 3.6 | 10.4 | 2.3 |
| 0.8 | 17.4 | 5.2 | 9.2 | 2.9 |
| 0.85 | 13.5 | 7.4 | 7.4 | 3.9 |
| 0.9 | 8.0 | 9.0 | 5.0 | 4.7 |
| 0.95 | 3.4 | 8.4 | 3.5 | 4.4 |
| 0.975 | 1.4 | 8.1 | 3.0 | 4.3 |
| 1 | 0 | 8.0 | 0 | 4.2 |

**Table S3: Simulated coupling strengths and energy splittings for various SiO2 layer positions.**

| *z_SiO2_/h_res_* | *Ω_r,1_*(meV) | *Ω_r,2_*(meV) | *g_1_*(meV) | *g_2_*(meV) |
| --- | --- | --- | --- | --- |
| 0.5 | 28.4±1.2 | 0 | 14.2±0.8 | 0 |
| 0.85 | 14.2±1.9 | 5.9±1.9 | 8.2±1.2 | 3.2±1.2 |
| 1 | 0 | 7.0±1.0 | 0 | 3.7±0.8 |

**Table S4: Experimental coupling strengths and energy splittings for various SiO2 layer positions.**

**Supplementary Figures**

**
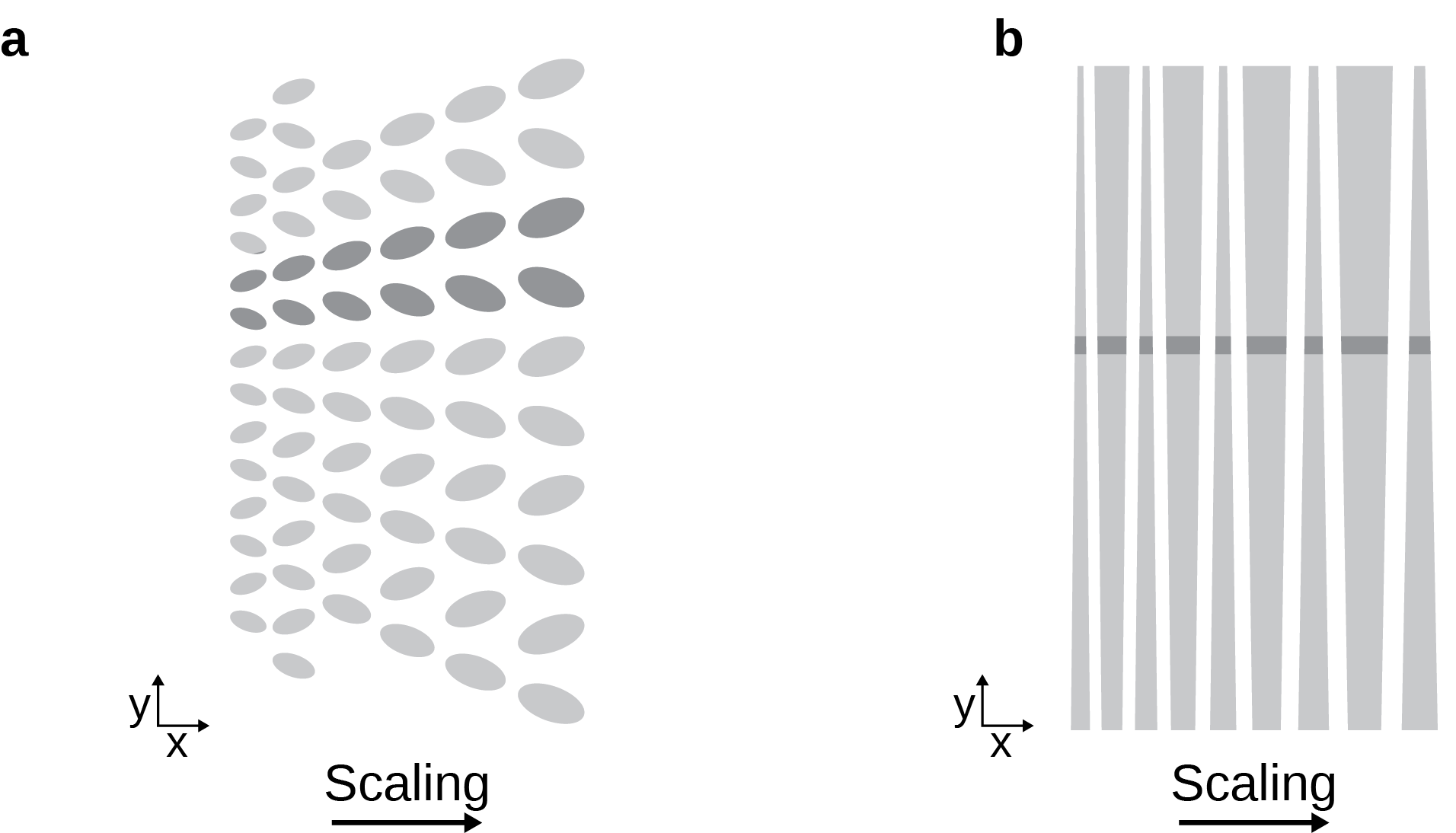
**

**Figure S1: Unit cell alignment. a.** Conventional high Q-factor scaling gradient design with scaling along the x-axis.^[3–5]^ In this design, the y-axis pitch increases between neighboring columns of resonators, disturbing the alignment within a row. This misalignment is evident in the highlighted row, where the y-center positions shift with scaling. **b.** Tapered-bar gradient with an infinitesimal y-axis pitch and scaling along the x-axis. This configuration enables perfect alignment of neighboring unit cells along the x-axis, a substantial improvement over previous designs.

**
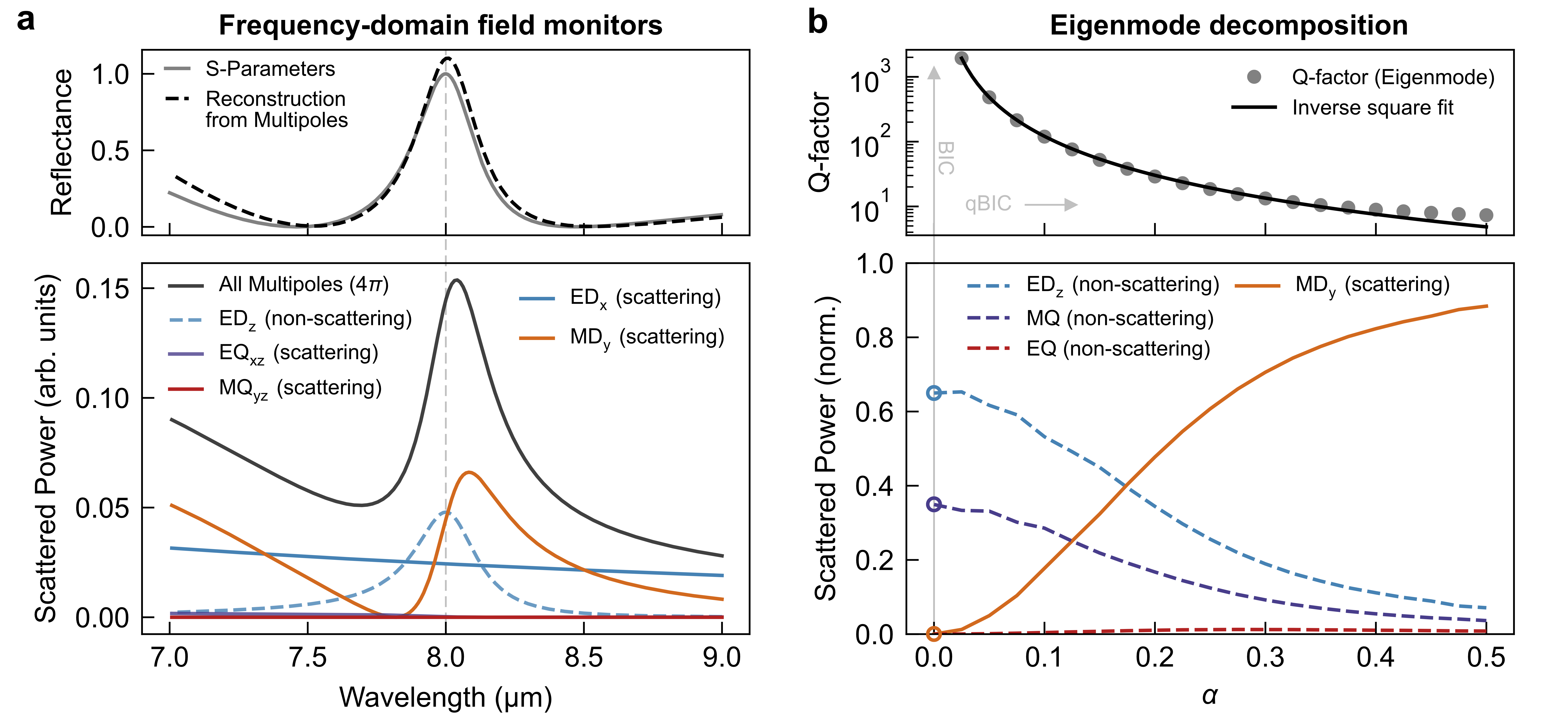
**

**Figure S2: Multipole decomposition. a**, Wavelength-resolved multipole analysis of a qBIC metasurface with asymmetry parameter $\alpha=0.2$, excited from the far field in x-polarization. The reflectance spectrum calculated via the respective S-parameter and its reconstruction from multipoles through near-field analysis show good agreement, validating the multipole analysis. The major contribution to the total scattered power is given by the magnetic dipole (MD), oriented along the y-direction, as well as a non-resonant background by an electric dipole (ED), induced by the excitation. Additionally, a resonant electric dipole along the z-axis forms, which cannot radiate to the far-field. Higher-order multipoles do not contribute significantly to the total scattered power. **b**, Multipole analysis of eigenmodes for different asymmetry parameters $\alpha$. The calculated Q-factors confirm the qBIC-typical inverse square behavior with respect to the asymmetry parameter. In the symmetric case $\alpha=0$, the BIC mode with diverging Q-factor is comprised of the non-scattering (dark) multiples ED in z-direction as well as a magnetic quadrupole (MQ), whereas the scattering contribution of the MD completely vanishes. Upon breaking the symmetry, a net in-plane MD forms, which couples to the far field and establishes a radiative loss-channel.

**
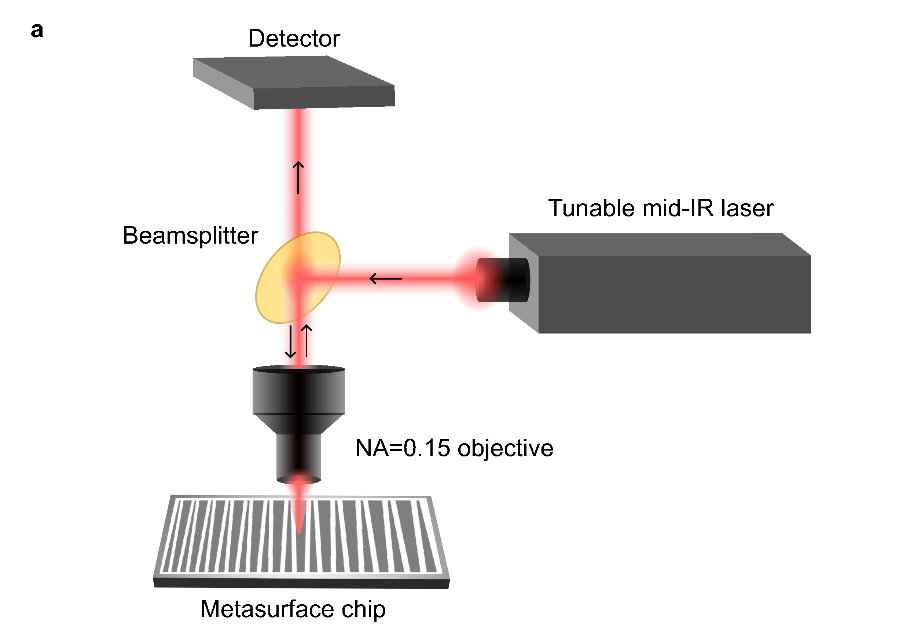
**

**Figure S3: Sketch of the measurement setup. a.** A tuneable mid-IR laser is guided via a dichroic mirror onto the sample through a high N.A. (𝑁.𝐴. = 0.15) objective. The reflected light is collected by the same objective and projected onto a 480x480 pixel detector. The lasers can be tuned in 2 cm^-1^ steps across the target spectral range. This results in an image being captured at each wavelength, allowing for hyperspectral imaging.


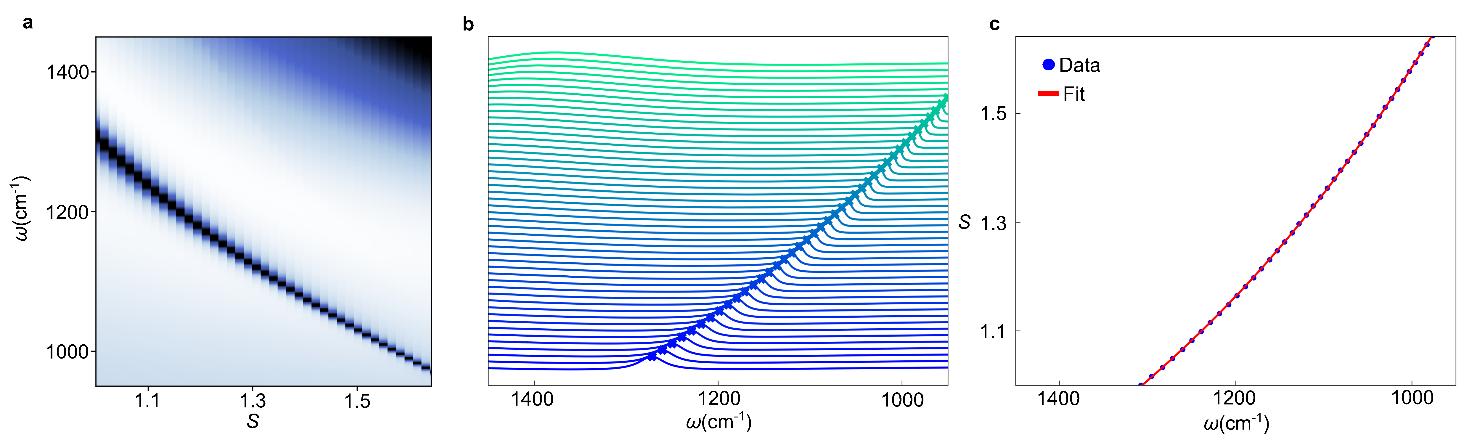


**Figure S4: Simulations of Si metasurface without SiO_2_. a.** Simulated reflectance spectra vs. scaling factor. **b**. Individual spectra across the target wavelength range. **c.** Fitted peaks vs. scaling factor, fitted with a cubic function of the form *ax^3^ + bx^2^ + cx + d* to capture the nonlinear behavior of the qBIC mode resonance position.


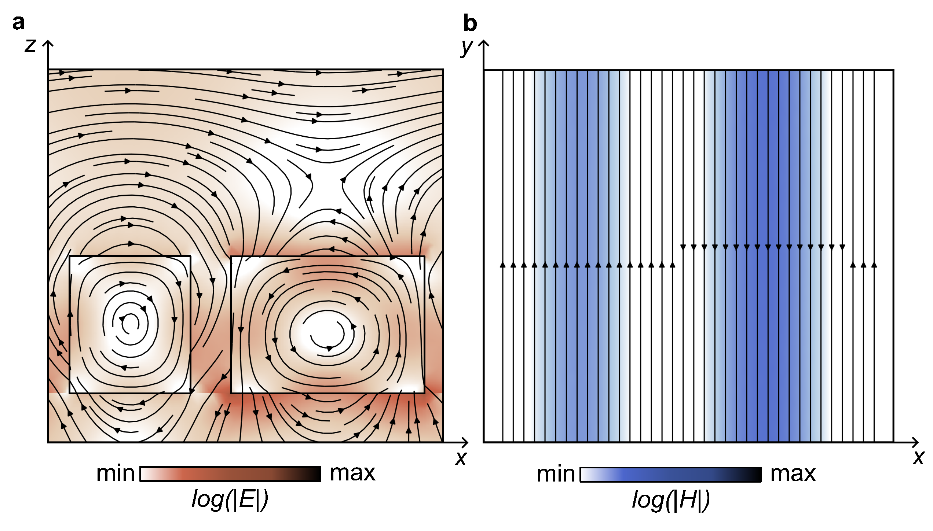


**Figure S5: Simulated *E*- and *H*-fields of pure Si tapered bar metasurface. a.** Cross-section of a single unit cell showing electric field lines forming a vortex inside the resonator at the qBIC resonance. **b.** Top view of a single unit cell showing the magnetic dipole that is generated at the qBIC resonance.


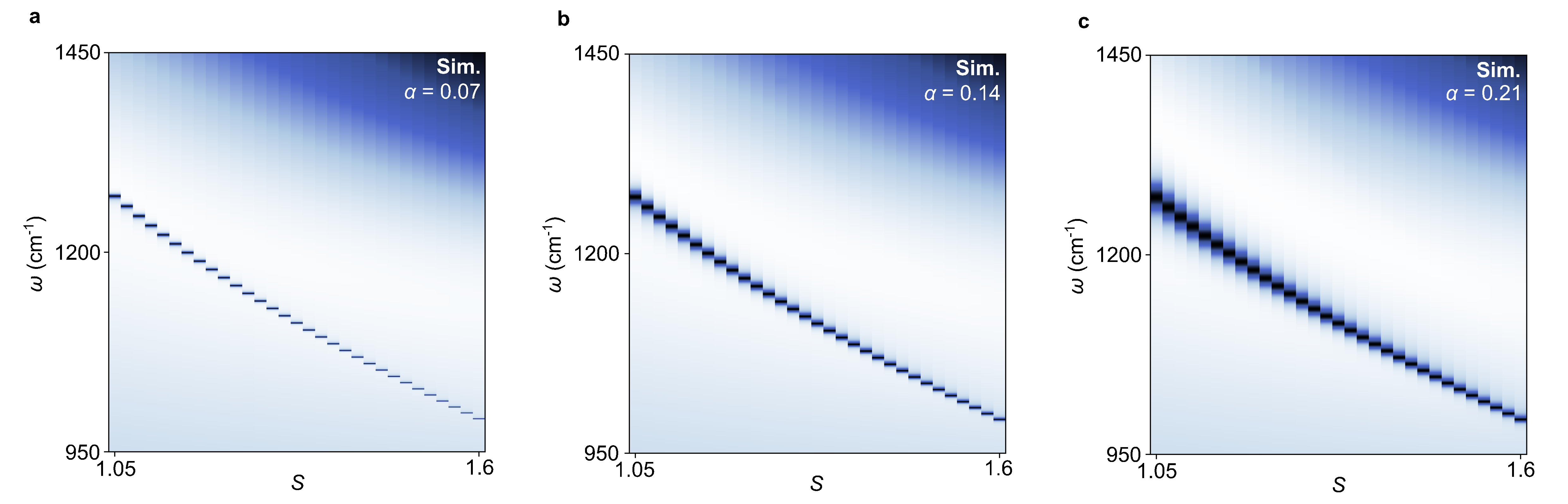


**Figure S6: Simulations of all-dielectric metasurface. a, b, c.** Simulated reflectance spectra vs. scaling factor for *α* = 0.07, *α* = 0.14 and *α* = 0.21.


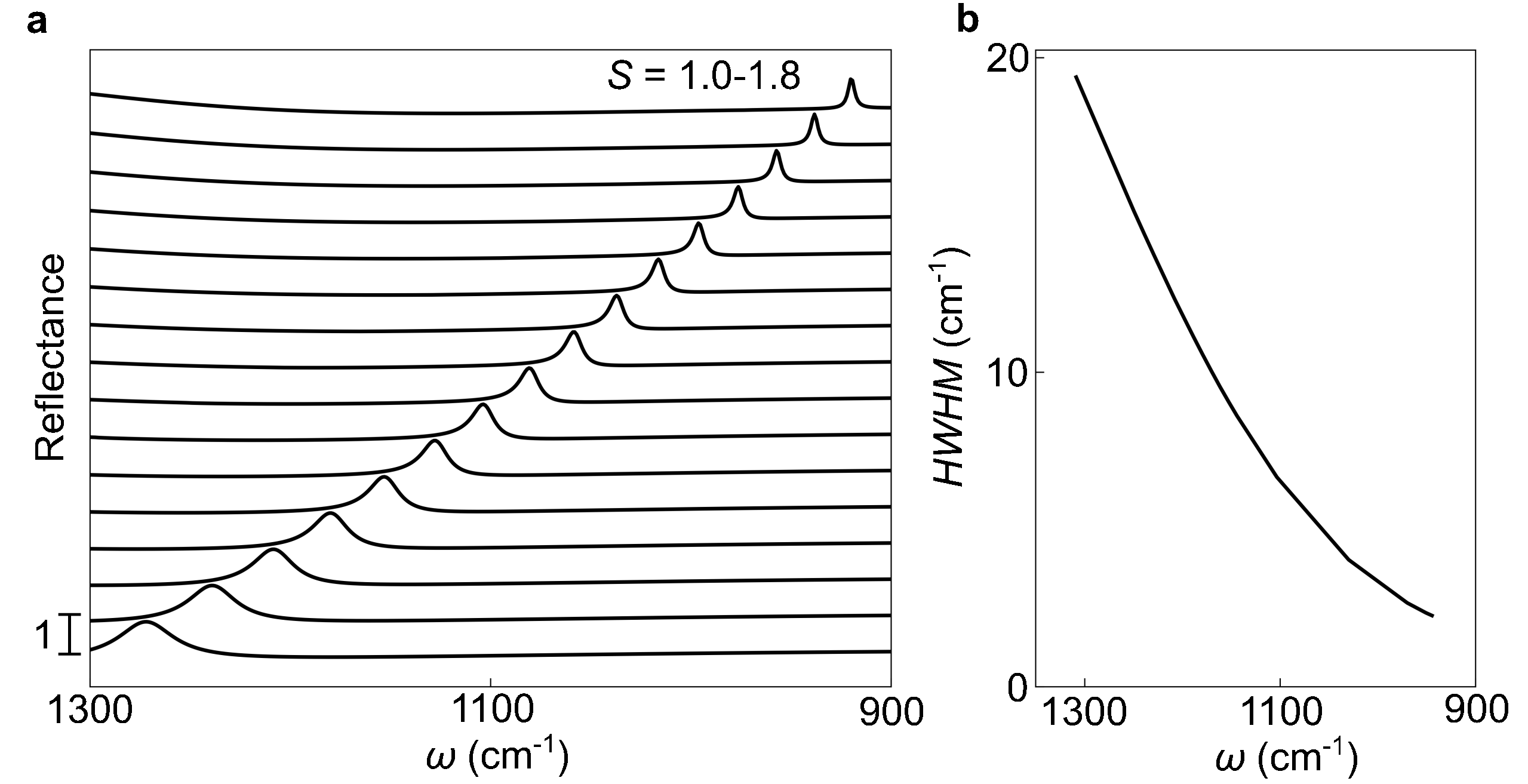


**Figure S7: HWHM of all-dielectric qBIC resonances. a** Simulated reflectance spectra vs. scaling factor for *α* = 0.21. **b** Fitted HWHM vs. resonance spectral position.


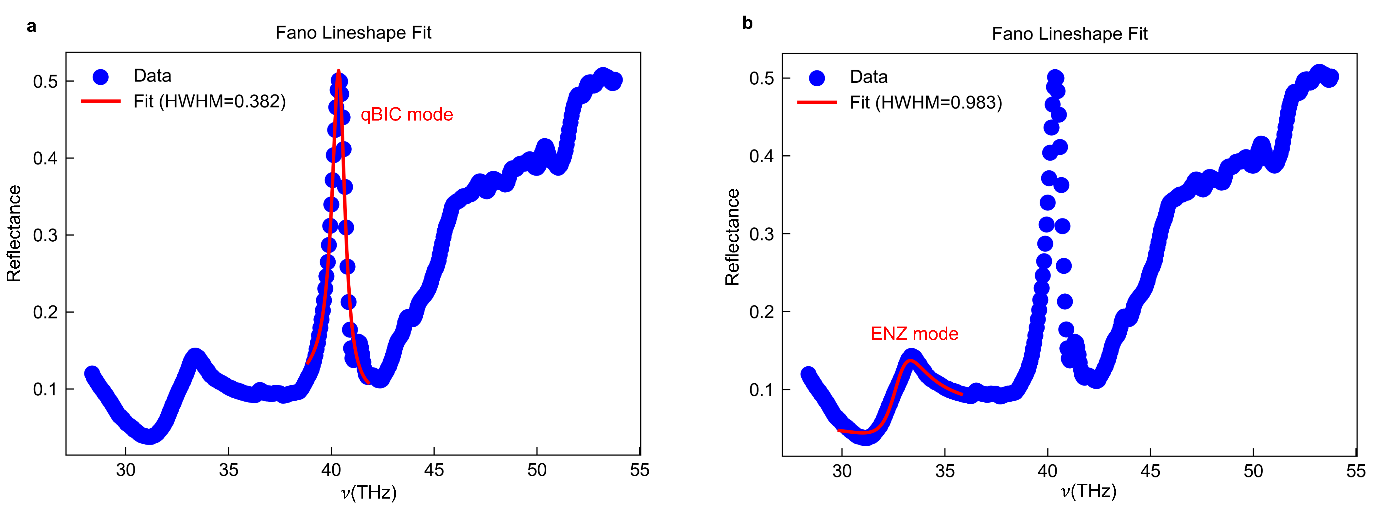


**Figure S8: Resonance linewidth fitting a, b.** Experimental fano lineshape fitting of qBIC and ENZ mode respectively (*h_SiO2_* = 114 nm). The fits yield a HWHM of 0.382 THz for the qBIC mode and 0.983 THz for the ENZ mode.


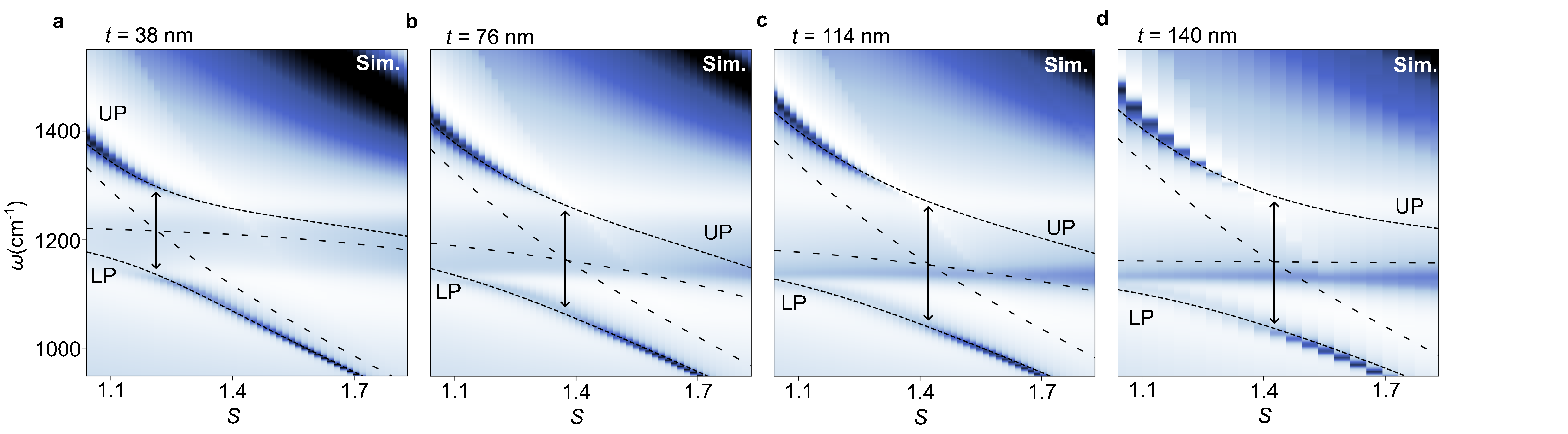


**Figure S9: Simulations of Si metasurface with SiO_2_ layer of varying thicknesses. a, b, c, d.** Simulated reflectance spectra vs. scaling factor for SiO_2_ thicknesses of *h_SiO2_=*38, 76, 114 and 140 nm respectively. The strong coupling fits are shown as dashed (UP and LP) and loosely dashed (qBIC, ENZ mode positions) curves.


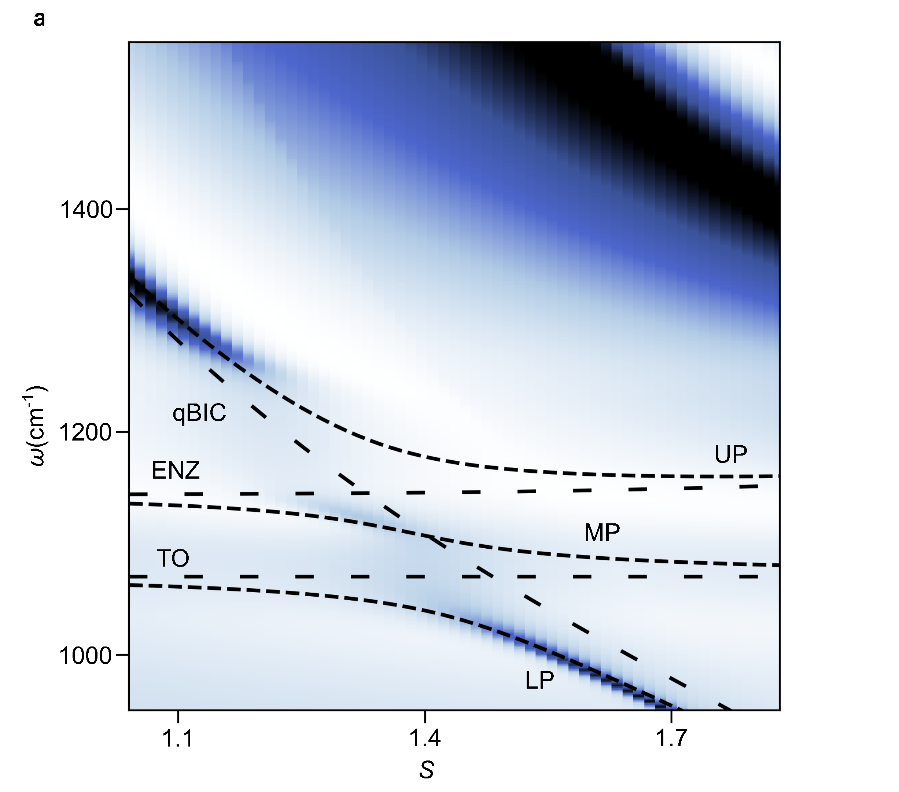


**Figure S10: Simulations of Si metasurface with SiO_2_ layer at position *z_SiO2_*=0.9*h_res_*. a.** Simulated reflectance spectra vs. scaling factor for ENZ position at ***z_SiO2_***=0.9*h_res_*. The strong coupling fits obtained from the triple coupled oscillator model are shown as dashed (UP, MP and LP) and loosely dashed (qBIC, EN, TO) curves.


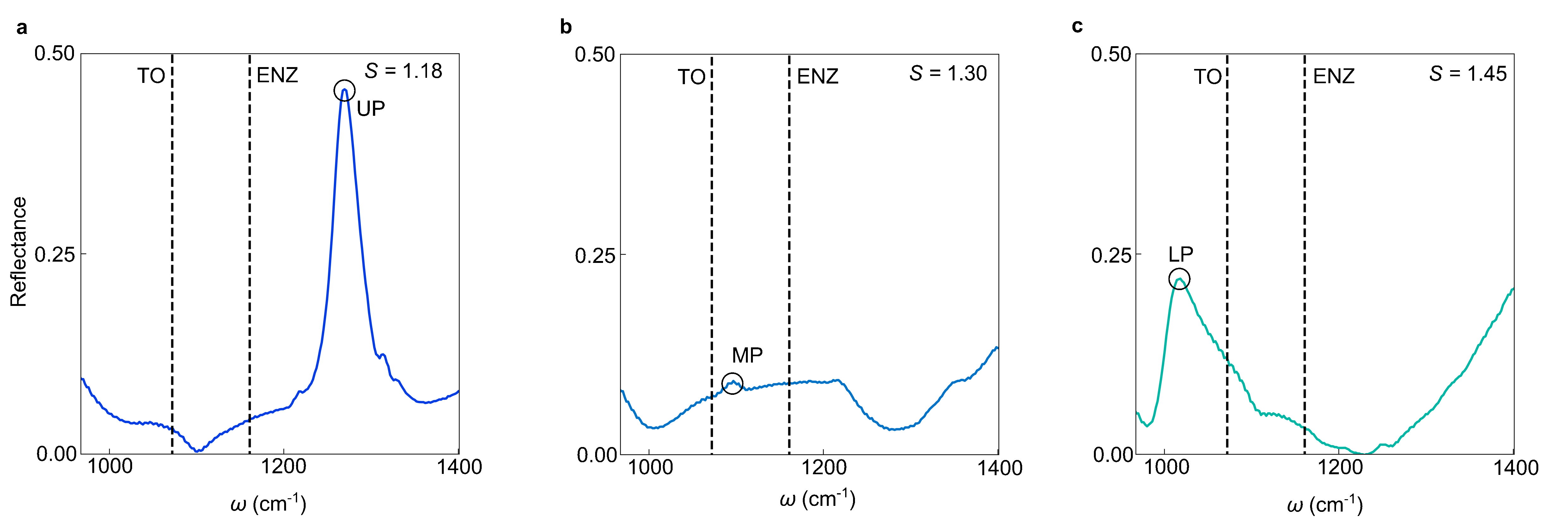


**Figure S11: Individual spectra of qBIC-ENZ-TO strong coupling. a, b, c** Spectra extracted from **Fig. 4e** for *S* = 1.18 (a), 1.30 (b) and 1.45 (c). The dashed lines mark the position of TO and ENZ mode respectively.


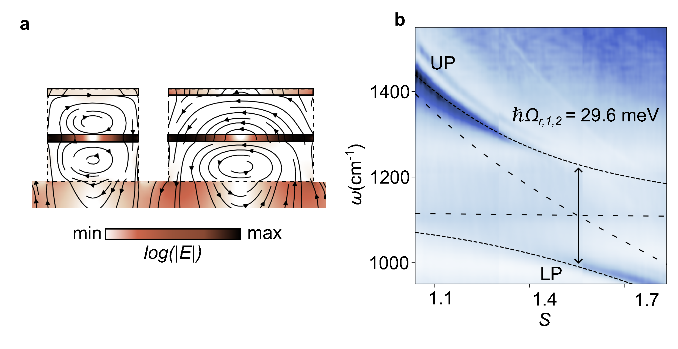


**Figure S12. Ultra-strong coupling of a qBIC mode to an ENZ mode and a TO phonon.** **a.** Simulated electric field distribution for a metasurface design featuring two SiO_2_ layers to maximize the combined qBIC–ENZ and qBIC–TO coupling. **b.** Experimental realization of ultra-strong coupling in the system shown in (a), exhibiting a Rabi splitting of $29.6 \mathrm{meV}$.


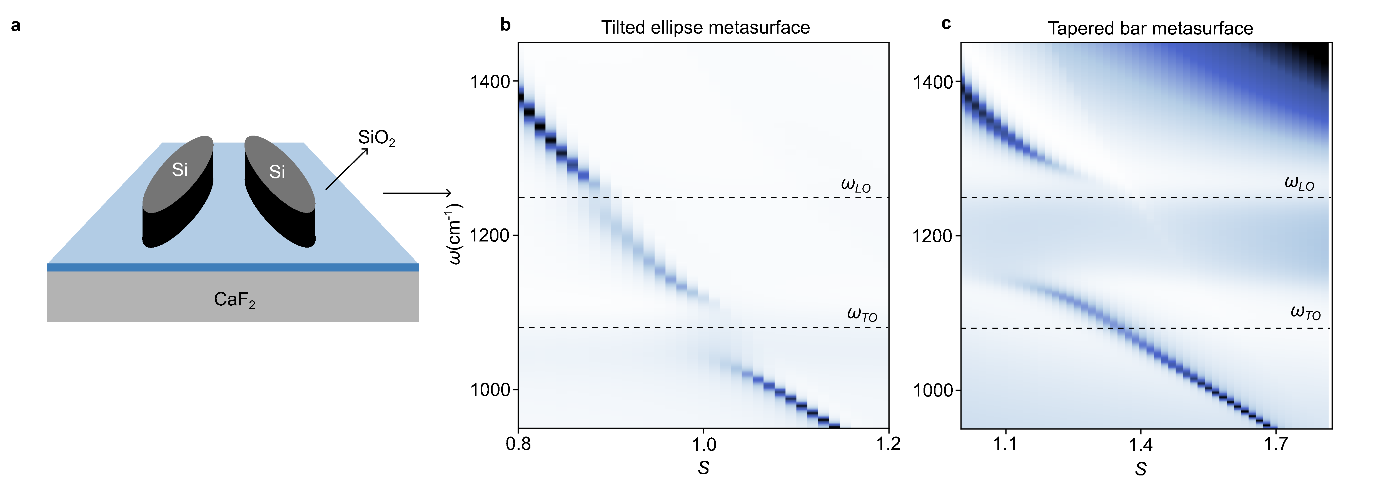


**Figure S13: Comparison of conventional approach and our proposed tapered bar design. a.** Sketch of a tilted ellipse metasurface consisting of Si ellipse resonators, with the resonant SiO_2_ layer placed on top of the substrate, similar to proposed designs.^[6]^ **b,c.** Simulated reflectance spectra of tilted ellipse metasurface shown in a and our proposed tapered bar metasurface for the same thickness of SiO_2_ (*h_SiO2_* = 80 nm). Compared to the tilted ellipse metasurface, the tapered bar metasurface shows much larger energy splitting around the ENZ mode, while fully supressing coupling between qBIC and TO phonon. *P_x_* = 4000 nm for *S* = 1.


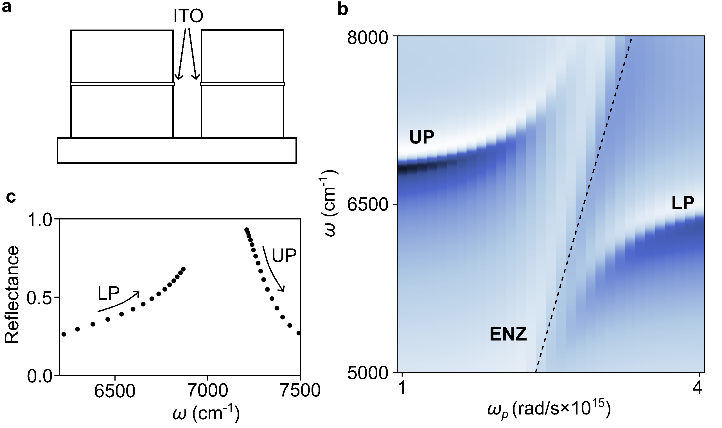


**Figure S14: Electrically tunable qBIC resonance with ITO. a** Tapered bar metasurface with ITO layer sandwiched in between high refractive index dielectrics similar to the tapered bar metasurface shown in **Fig. 1**. **b** Simulation of the structure shown in (a) using a layer of *t_ITO_* = 5 nm while varying the plasma frequency between 1×10^15^ and 4×10^15^ rad/s. The spectral shift of the plasma frequency causes a spectral shift of the ENZ mode and thus enables tunable splitting of the qBIC resonance. Values for the permittivity of ITO were taken from literature^[7]^. **c** Positions of the maxima of UP and LP extracted from (b). The modulation of the carrier density enables broadband tunability of the qBIC resonance (around 10-15% frequency shift).


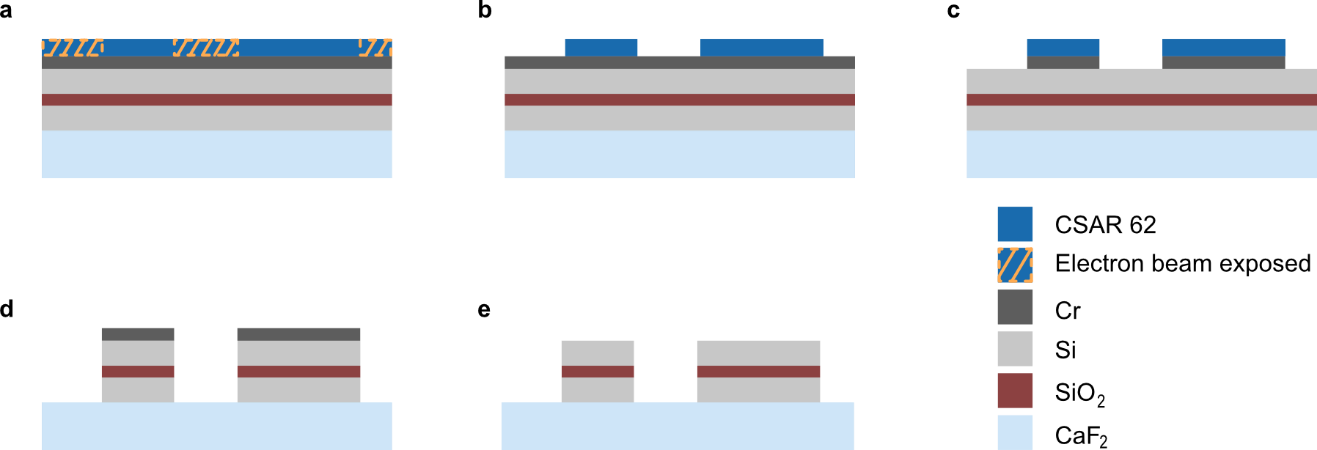


**Figure S15: Fabrication Workflow** **a.** Stack of Si, SiO_2_, Si, Cr and CSAR 62 resist on a CaF_2_ substrate after electron beam exposure in gap areas. **b.** Development. **c.** Cr reactive ion etching. **d.** Reactive ion etching of Si, SiO_2_ and again Si. **e.** Cr mask removal in final dry etching step.

**References**

[1] E. A. Gurvitz, K. S. Ladutenko, P. A. Dergachev, A. B. Evlyukhin, A. E. Miroshnichenko, A. S. Shalin, *Laser & Photonics Reviews* **2019**, *13*, 1800266.

[2] P. D. Terekhov, V. E. Babicheva, K. V. Baryshnikova, A. S. Shalin, A. Karabchevsky, A. B. Evlyukhin, *Phys. Rev. B* **2019**, *99*, 045424.

[3] A. Aigner, T. Weber, A. Wester, S. A. Maier, A. Tittl, *Nat. Nanotechnol.* **2024**, *19*, 1804.

[4] P. Jangid, F. U. Richter, M. L. Tseng, I. Sinev, S. Kruk, H. Altug, Y. Kivshar, *Advanced Materials* **2024**, *36*, 2307494.

[5] F. U. Richter, I. Sinev, S. Zhou, A. Leitis, S.-H. Oh, M. L. Tseng, Y. Kivshar, H. Altug, *Advanced Materials* **2024**, *36*, 2314279.

[6] L. Yue, P. Xie, S. Shen, Q. Ding, H. Zhang, W. Wang, *Physical Review B* **2024**, *109*, 205405.

[7] A. Minenkov, S. Hollweger, J. Duchoslav, O. Erdene-Ochir, M. Weise, E. Ermilova, A. Hertwig, M. Schiek, *ACS Appl. Mater. Interfaces* **2024**, *16*, 9517.
